# Entropy Analysis for the Evaluation of Respiratory Changes Due to Asbestos Exposure and Associated Smoking

**DOI:** 10.3390/e21030225

**Published:** 2019-02-27

**Authors:** Paula M. Sá, Hermano A. Castro, Agnaldo J. Lopes, Pedro L. Melo

**Affiliations:** 1 Institute of Biology and Faculty of Engineering, Biomedical Instrumentation Laboratory, State University of Rio de Janeiro, Rio de Janeiro 20550-900, Brazil; 2 Oswaldo Cruz Foundation, National School of Public Health, Rio de Janeiro 21040-900, Brazil; 3 Pedro Ernesto University Hospital, State University of Rio de Janeiro, Rio de Janeiro 20550-900, Brazil; 4 Rehabilitation Sciences Post-Graduate Programme, Augusto Motta University Centre, Rio de Janeiro 21041-020, Brazil; 5 Laboratory of Clinical and Experimental Research in Vascular Biology, State University of Rio de Janeiro, Rio de Janeiro 20550-900, Brazil

**Keywords:** sample entropy, recurrence period density entropy, respiratory system complexity, asbestos-exposed workers, smoking, diagnostic, early diagnosis, respiratory diseases, forced oscillation technique, respiratory impedance

## Abstract

Breathing is a complex rhythmic motor act, which is created by integrating different inputs to the respiratory centres. Analysing nonlinear fluctuations in breathing may provide clinically relevant information in patients with complex illnesses, such as asbestosis. We evaluated the effect of exposition to asbestos on the complexity of the respiratory system by investigating the respiratory impedance sample entropy (SampEnZrs) and recurrence period density entropy (RPDEnZrs). Similar analyses were performed by evaluating the airflow pattern sample entropy (SampEnV’) and recurrence period density entropy (RPDEnV’). Groups of 34 controls and 34 asbestos-exposed patients were evaluated in the respiratory impedance entropy analysis, while groups of 34 controls and 30 asbestos-exposed patients were investigated in the analysis of airflow entropy. Asbestos exposition introduced a significant reduction of RPDEnV’ in non-smoker patients (p < 0.0004), which suggests that the airflow pattern becomes less complex in these patients. Smoker patients also presented a reduction in RPDEnV’ (p < 0.05). These finding are consistent with the reduction in respiratory system adaptability to daily life activities observed in these patients. It was observed a significant reduction in SampEnV’ in smoker patients in comparison with non-smokers (p < 0.02). Diagnostic accuracy evaluations in the whole group of patients (including non-smokers and smokers) indicated that RPDEnV’ might be useful in the diagnosis of respiratory abnormalities in asbestos-exposed patients, showing an accuracy of 72.0%. In specific groups of non-smokers, RPDEnV’ also presented adequate accuracy (79.0%), while in smoker patients, SampEnV’ and RPDEnV’ presented adequate accuracy (70.7% and 70.2%, respectively). Taken together, these results suggest that entropy analysis may provide an early and sensitive functional indicator of interstitial asbestosis.

## 1. Introduction

In recent years, there has been an increasing interest in the use of nonlinear analysis to characterize respiratory patterns [1,2,3,4,5,6,7,8]. In particular, the complexity of the respiratory signals can be used as a measure of changes in the respiratory system due to ageing and diseases [9]. Such analyses may complement information obtained by traditional pulmonary function exams and increase our understanding of respiratory disorders.

Asbestosis is an interstitial lung disease that is associated with exposure to asbestos, the severity of which is proportional to the duration and intensity of the exposure time [10,11,12]. Traditional pulmonary function exams in these patients usually reveal a restrictive ventilatory disorder. Previous studies in humans and small animals have provided evidence that the limitation of expiratory flow associated with small airway disease could indicate an early manifestation of asbestosis [13]. In practice, several of these patients are smokers, a habit that has been reported to introduce an additional obstructive alteration in these patients [10,14]. Worldwide, it is estimated that more than 100,000 deaths/year are caused by asbestosis [15]. Considering the rising number of cases of asbestos-related diseases, the International Labour Organization and World Health Organization has recommended that special attention should be paid to the elimination of asbestos-related diseases [16]. Thus, the early detection of asbestosis followed by management is essential for providing better healthcare support to these patients.

Recent studies from our group have provided evidence that the airflow patterns in asthma [7] and chronic obstructive pulmonary disease (COPD) [17], as well as the respiratory impedance patterns in asthmatic patients [18], become less complex than in normal subjects. Similar reductions have recently been observed by other researchers [19]. It is possible that the exposure to asbestos may also generate changes in the complexity of the respiratory system, which may be reflected in the respiratory impedance and airflow measurements. In addition, several of these patients are smokers, which may also introduce important changes in respiratory system complexity. These evaluations have strong potential to aid in the clinical diagnosis of respiratory alterations in asbestosis. The measurements are simple and require only passive cooperation with no forced expiratory manoeuvres. Although these analyses present a high potential to improve our knowledge concerning the biomechanical changes, as well as the respiratory exams in asbestosis, no previous study has closely investigated this matter. Only a recent conference paper describing the initial results of our group studying airflow pattern complexity is available [20].

Based on the abovementioned considerations, the main objectives of this validation study were as follows: (1) to evaluate the ability of entropy analysis of the respiratory impedance and airflow signals to describe the abnormal respiratory effects of the early stage of asbestosis, and (2) to evaluate the clinical diagnostic use of complexity measures in the general population of patients and specific groups of non-smoker and smoking patients.

## 2. Materials and Methods

The study was approved by the Ethics Committee of the Pedro Ernesto University Hospital and was registered at ClinicalTrials.gov (identifier: NCT02280343) and conducted in accordance with the Declaration of Helsinki. All the volunteers signed a written informed consent before their inclusion in the study. Patients were from the Workers’ Health and Human Ecology Study Center (CESTEH) at the National School of Public Health Sergio Arouca (ENSP), Oswaldo Cruz Foundation (FioCruz). Radiological categorization was performed according to the International Labour Organization [21].

### 2.1. Volunteers

#### 2.1.1. Respiratory Impedance Complexity

Sixty-eight subjects were analysed in this study. The control group (CG) consisted of 34 adult subjects with a normal spirometric exam who did not have a history of smoking or pulmonary disease. Thirty-four subjects who were exposed to asbestos were also studied. This group was composed of exposed volunteers with radiographs categorized as 0/0 (n = 29) and volunteers with radiological categories of 0/1 or 1/1 (n = 5) [21]. The subjects were workers exposed to asbestos but without a clinical diagnosis of asbestosis. Smoking was not an exclusion criterion, so that 12 patients were smokers. 

#### 2.1.2. Airflow Pattern Complexity

The studied sample consisted of sixty-four volunteers, 34 of whom belonged to the control group. Thirty asbestos-exposed patients were also studied, and smoking was not an exclusion criterion, so that 12 patients were smokers. Twenty-five patients presented radiographs categorized as 0/0, and five were categorized as 0/1 or 1/1 [21]. Similar to the evaluation of respiratory impedance, the workers were exposed to asbestos but without a clinical diagnosis of asbestosis.

### 2.2. Pulmonary Function Analysis

The volunteers were evaluated in the Biomedical Instrumentation Laboratory at the State University of Rio de Janeiro (UERJ). To avoid changes in bronchial tone, the exams were performed in the following order: forced oscillation technique, spirometry, and plethysmography. 

Forced oscillations was measured at 5 Hz using a device developed in our laboratory [22]. During the measurements, the subject remained under spontaneous ventilation while a low-pressure sinusoidal signal (2.0 cm H_2_O peak-to-peak amplitude) was applied by the instrument to the subject’s respiratory system. The instrument allows the evaluation of the respiratory system impedance (Zrs) from signals coming from a pressure transducer and a pneumotachograph placed close to the individual’s mouth. The resulting pressure (P) and airflow (V’) signals are used to obtain the within-breath impedance module (Zrs = P/V’). 

Spirometric parameters were expressed as absolute values and as a percentage of the predicted values (% of predicted), and the reference values were obtained from the equations of Pereira et al. [23]. These exams included the forced expiratory volume in the first second (FEV_1_), forced vital capacity (FVC), FEV_1_/FVC ratio, and forced expiratory flow (FEF) between 25% and 75% of the FVC (FEF/FVC) ratio. 

Plethysmographic exams used the reference values based on the equations described by Neder et al. [24]. They were performed with a constant volume and variable pressure plethysmograph (HD CPL nSpire Health Ltd., Hertford, UK). The evaluated parameters were the total lung capacity (TLC), functional residual capacity (FRC) and residual volume (RV), as well as their relationships (RV/TLC and FRC/TLC). Airway resistance (Raw) was also measured. 

### 2.3. Complexity Analysis

Respiratory impedance and airflow signals were measured over 120 s, and these signals were digitized at a sampling rate of 16 Hz with a 12-bit resolution. The first sixty seconds were discarded to minimize any influence that increased familiarity with the study apparatus. Low frequency trends were removed [25]. Signals with distortions due to sneezes, coughs or swallows were discarded. As a result, time series of 60 s were analysed.

To quantify the respiratory impedance and airflow time series complexity, the sample entropy (SampEn) [26] was initially used. This analysis evaluates the time-dependent structure of the input signal and returns a value between 0 and approximately 2, which represents the predictability of future values in the time series based on previous values. A SampEn value of zero would correspond to a sine-wave signal that has a high degree of short and long-term predictability, describing a low complexity system. On the other hand, if a completely random signal, such as pure white Gaussian noise, is assessed with SampEn, the value returned would be closer to 2 based on the fact that, in a completely random signal, future values in the time series are independent and unpredictable based on previous values. This result arises from highly complex systems. This analysis has been successfully used in previous studies conducted by our group [7,17,18,27] and widely adopted in the fields of heart rate variability [28] and neuroscience [29]. SampEn (m, r) values for all data sets were calculated using m = 2 and r = 20% of the SD of the studied time series. We set m = 2 taking into consideration our record length (N = 960) and following the recommendation of Pincus and Goldberger [30] to use 10^m^ to 30^m^ points for analysis. This also allows direct comparisons with previous results [25] from our laboratory [17,18] and other researchers [19].

We also evaluated the entropy of the recurrence period density (RPDEn). This parameter reflects the signal regularity describing the uncertainty in the trajectory period of the attractor constructed based on the analysed series, thus increasing with the irregularity of the signal [31]. Using the CRP Toolbox for MATLAB [31], RPDEn was calculated with an embedding dimension of three [32] and a time lag of 12 samples (750 ms) and 10 samples (625 ms) for respiratory impedance and airflow, respectively. These values were chosen as the average time lag at which the autocorrelation functions first nears 1/e [32]. The embedding dimension was determined based on previous studies in the area of respiratory physiology [33,34,35] and according to Liebert et al. [36], which showed that an adequate phase-space reconstruction may be obtained with a percentage of false nearest neighbors between 10 and 15%.

### 2.4. Statistics

Initially, the characteristics of the sample distribution were evaluated using the Shapiro Wilk test. Then, comparisons among groups were performed using Tukey’s test to analyse normally distributed data; conversely, a non-parametric analysis (Mann-Whitney test) was performed for the non-normally distributed data. Results with p < 0.05 were considered to be statistically significant, and a commercial software package (Origin® 8.0, Microcal Software Inc., Northampton, MA, USA) was used in these analyses. We initially evaluated if asbestos exposure changes the entropy of respiratory time-series, and then performed a preliminary analysis evaluating if the smoking habit further exacerbates the effect of asbestos exposure.

The associations among the entropy and pulmonary function parameters were evaluated using Spearman’s correlation coefficients. These analyses were performed using GraphPad Prism (version 7.0 for Windows, GraphPad Software, San Diego, CA, USA). 

The clinical potential of the complexity indexes in the detection of the early respiratory alterations in asbestosis was evaluated using receiver operation characteristic (ROC) curves. The values of sensitivity, specificity, and area under the curve (AUC) were obtained based on the optimal cut-off point, as determined by the ROC curve analysis. The optimal cut-off point is chosen to balance the highest values of sensitivity and specificity. These analyses were performed using MedCalc 12 (MedCalc Software, Mariakerke, Belgium). In this study, following the work of Goedhart et al. [37], an AUC of 0.7 was considered a good cut-off value as a useful discriminator for clinical use. There were a relatively small number of patients in the studied groups. To minimize this statistical problem, we employed leave-one-out cross-validation (LOOCV) analyses, which were performed according to Witten [38]. 

## 3. Results

### 3.1. Demographics, Pulmonary Function and Effect of Smoking in the Respiratory Impedance Analysis

Biometric and spirometric characteristics of the groups evaluated in this analysis are presented in Table 1.

### 3.2. Complexity Analysis of the Respiratory Impedance Signal

**Figure 1:**
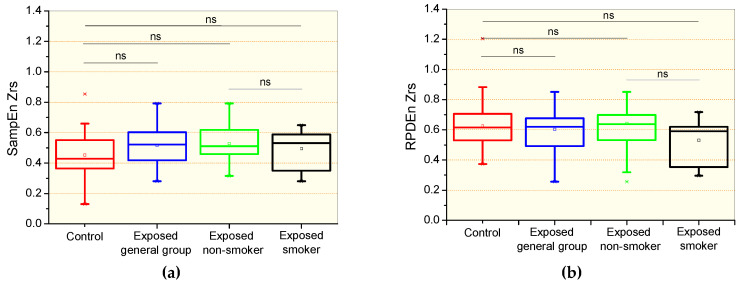
Sample entropy of the respiratory impedance (SampEnZrs, **a**); recurrence period density entropy of the respiratory impedance signal (RPDEnZrs, **b**) in controls, the general patient group, including non-smokers and smokers, patients exposed to asbestos non-smokers, and patients exposed and smokers. The top and the bottom of the box plot represent the 25th- to 75th-percentile values, while the circle represents the mean value, and the bar across the box represents the 50th-percentile value. The whiskers outside the box represent the 10th-to 90th-percentile values. Ns = non-significant.

The comparative analysis of the respiratory impedance complexity is shown in Figure 1. There were no significant differences in the comparison between the control and the general patient group, including non-smokers and smokers, and specific groups of non-smokers and smokers patients for the sample entropy of Zrs (SampEnZrs, Figure 1a) and the recurrence period density entropy of Zrs (RPDEnZrs, Figure 1b). Smoking introduced non-significant reductions in SampEnZrs (Figure 1a) and RPDEnZrs (Figure 1b).

**Table 2:** Effect of smoking in exposed subjects in the respiratory impedance analysis. Significant changes are described in bold.

	Non-Smokers (n = 22)	Smokers (n = 12)	p
Spirometry			
FVC (L)	2.65 ± 0.69	3.08 ± 0.51	0.07
FVC (%)	85.5 ±13.1	93 ± 20.6	0.37
FEV_1_ (L)	2.07 ± 0.50	2.04 ± 0.45	0.87
FEV_1_ (%)	84.6 ± 14.2	78.4 ± 15.4	0.24
FEV_1_/FVC	78.6 ± 6.76	66.7 ± 12.4	**<0.004**
FEF_25–75%_ (L)	2.14 ± 0.75	1.36 ± 0.81	**<0.009**
FEF_25–75%_ (%)	90.1 ± 32.1	51.3 ± 29.2	**<0.004**
FEF/FVC	106.3 ± 38.30	57.8 ± 35.5	**<0.002**
Plethysmography			
RV (L)	1.90 ± 0.95	2.80 ± 1.05	**<0.02**
RV (%)	116.7 ± 46.87	155.1 ± 82.24	0.09
TLC (L)	4.63 ± 1.22	5.90 ± 0.91	**<0.004**
TLC (%)	98.6 ±18.0	114.7 ± 30.16	0.09
RV/TLC (L)	38.2 ± 10.4	46.5 ± 10.3	**<0.04**
Raw (cmH_2_O/L/s)	5.63 ± 10.4	5.17 ± 5.15	0.88
Raw (%)	420.9 ± 776.6	372.1 ± 424.0	0.84
sGaw (L/s/cmH_2_O/L)	0.23 ± 0.37	0.09 ± 0.06	0.20
sGaw (%)	90.97 ± 141.8	48.4 ± 36.4	0.33
Forced Oscillation			
Zm (cmH_2_O/L/s)	4.97 ± 1.77	5.06 ± 2.26	0.90

Values are presented as the mean ± standard deviation; significant correlations are presented in bold; FVC (L): forced vital capacity (litres); FVC (%): forced vital capacity (percentile values); FEV_1_ (L): forced expiratory volume in the first second (litres); FEV_1_ (%pred): forced expiratory volume in the first second (percentile of the predicted values); FEF_25–75%_: forced expiratory flow between 25% and 75% of the FVC; RV: residual volume (litres); TLC: total lung capacity; raw: airway resistance; sGaw: specific airway resistance; Zm: respiratory impedance modulus.

### 3.3. Demographics, Pulmonary Function and Effect of Smoking in the Airflow Analysis

Table 3 presents the biometrical and spirometric characteristics of the volunteers evaluated in this stage of the study. Exposed subjects presented a reduced FVC (L) and FEV_1_. 

Table 4 describes the pulmonary function changes due to smoking habits in asbestos-exposed patients. Smoking introduced significant changes in the spirometric indexes of small airway obstruction (FEF25-75%) and the plethysmographic indexes of residual volumes (RV).

### 3.4. Complexity Analysis of the Airflow Signal

**Figure 2:**
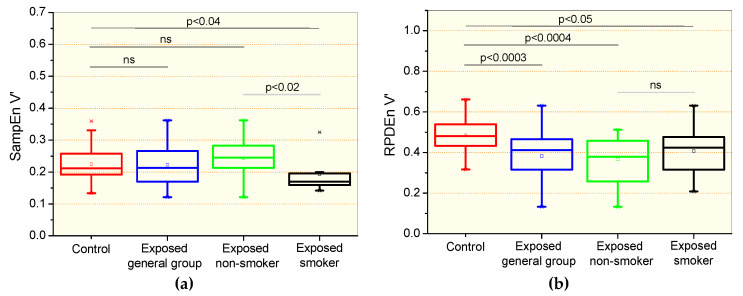
Sample entropy of the airflow signal (SampEnV’, **a**) and recurrence period density entropy of the airflow signal (RPDEnV’, **b**) in controls, the general group of patients including smokers and non-smokers, patients exposed to asbestos who were non-smokers, and patients exposed and smokers. The top and the bottom of the box plot represent the 25th- to 75th-percentile values, while the circle represents the mean value, and the bar across the box represents the 50th-percentile value. The whiskers outside the box represent the 10th-to 90th-percentile values. Ns = non-significant.

Changes in entropy parameters observed in the studied groups are presented in Figure 2. There were no significant differences in the comparisons between the control and the general patient group and non-smoker group for the sample entropy of the airflow signal (SampEnV’, Figure 2a). Significant reductions were observed in the SampEnV’ of the smoking group in comparison with the control group (p < 0.04) and non-smoking exposed patients (p < 0.02). Significant reductions in the recurrence period density entropy of the airflow (RPDEnV’) were observed in all of the studied asbestos exposed groups in comparison with controls (Figure 2b). Smoking in exposed patients introduced significant reductions (p < 0.02) in SampEnV’ (Figure 2a) and non-significant changes in RPDEnV’ (Figure 2b).

### 3.5. Correlation Among the Entropy Analysis and Pulmonary Function Exams

Considering the associations between the entropy parameters and those used in the pulmonary function analysis (Table 5), we observed that SampEnZrs exhibited a significant inverse correlation with the respiratory impedance modulus. RPDEnZrs showed a direct significant correlation with FEF_25–75%_ and an inverse relation with the respiratory impedance modulus.

### 3.6. Diagnostic Accuracy

#### 3.6.1. Analysis including Non-Smokers and Smokers Patients (the General Patient Group)

**Figure 3:**
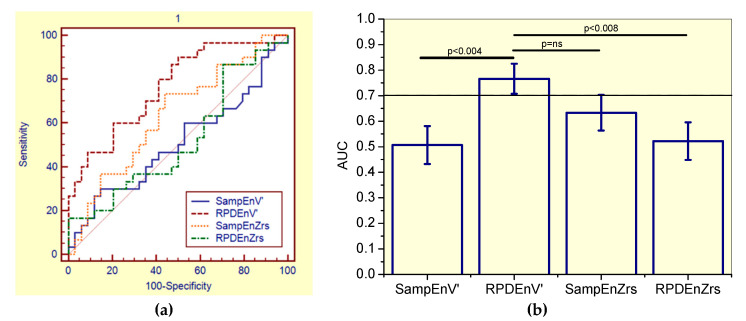
(**a**) Receiver-operating characteristic curves associated with the values presented in Table 6, including smoker and non-smoker patients. (**b**) Comparative analysis of the ability of the studied entropy indexes to diagnose initial respiratory changes in asbestos-exposed workers. The dotted horizontal line describes the minimal value of AUC considered adequate for clinical use. Ns = non-significant.

This evaluation showed that SampEnV’ was not adequate for clinical use, while RPDEV’ presented adequate accuracy (Table 6). The associated receiver-operating characteristic curves are presented in Figure 3a.

By comparing the ability of all the studied entropy indexes to identify initial changes in the general patient group of asbestos-exposed workers (Figure 3), the RPDEnV’ showed a significantly higher AUC than SampEnV’ (p < 0.004) and RPDEnZrs (p < 0.008). LOOCV analyses performed in RPDEnV’ resulted in an adequate accuracy for clinical use (AUC = 0.72, Se = 0.64, Sp = 0.64, cut-off = 0.490).

#### 3.6.2. Analysis including only Non-Smoker Patients

**Figure 4:**
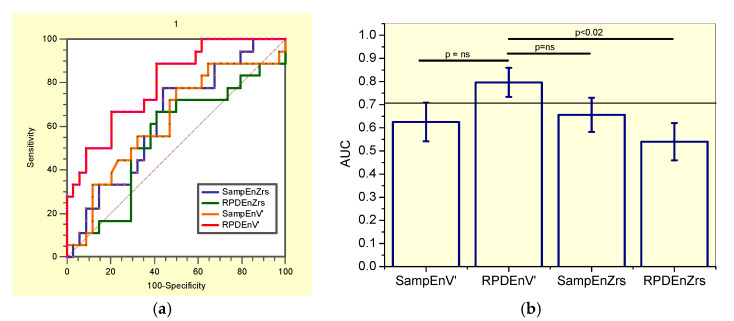
(**a**) Receiver-operating characteristic curves associated with the values presented in Table 7, describing analysis in non-smoker patients. (**b**) Comparative analysis of the ability of the studied entropy indexes to diagnose initial respiratory changes in non-smoker asbestos-exposed workers. The dotted horizontal line describes the minimal value of AUC considered adequate for clinical use. Ns = non-significant.

This evaluation showed that SampEnZrs, RPDEZrs, and SampEnV’ were not adequate for clinical use in non-smoker patients, while RPDEnV’ presented adequate accuracy (Table 7). The correspondent receiver-operating characteristic curves are described in Figure 4a.

The comparisons of the ability of all the studied entropy indexes to identify initial changes in non-smokers asbestos-exposed workers (Figure 4b), showed that RPDEnV’ showed a significantly higher AUC than RPDEnZrs (p < 0.02). LOOCV analyses performed in RPDEnV’ resulted in an adequate accuracy for clinical use (AUC = 0.79, Se = 0.89, Sp = 0.61, cut-off = 0.493).

#### 3.6.3. Analysis including only Smoker Patients

**Figure 5:**
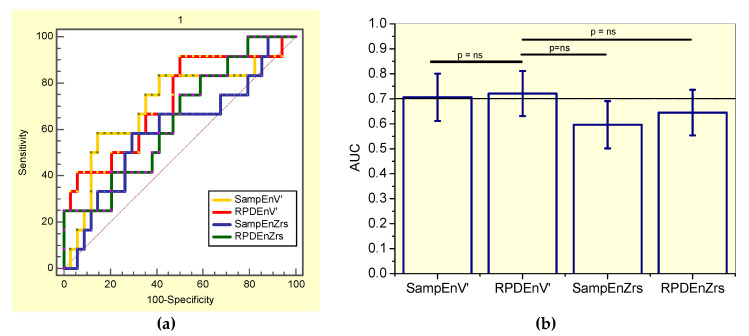
(**a**) Receiver-operating characteristic curves associated with the values presented in Table 8, describing analysis in smoker patients. (**b**) Comparative analysis of the ability of the studied entropy indexes to diagnose initial respiratory changes in smoker asbestos-exposed workers. The dotted horizontal line describes the minimal value of AUC considered adequate for clinical use. Ns = non-significant.

It was observed that SampEnZrs, RPDEZrs were not adequate for clinical use in smoker patients, while SampEnV’ and RPDEnV’ presented adequate diagnostic accuracy (Table 8). The associated receiver-operating characteristic curves are presented in Figure 5a. Figure 5b compares the ability of all the studied entropy indexes to identify initial changes in smoker asbestos-exposed workers. The RPDEnV’ showed the highest AUC, however this was not significantly higher than that observed in the other entropy indexes. LOOCV analyses resulted in adequate diagnostic accuracy for SampEnV’ (AUC = 0.71, Se = 0.67, Sp = 0.59, cut-off = 0.178) and RPDEnV’ (AUC = 0.70, Se = 0.75, Sp = 0.50. cut-off = 0.468).

## 4. Discussion

To the best of our knowledge, this study is the first to systematically evaluate the ability of the entropy analysis to describe the well-known respiratory effects of exposition to asbestos in non-smoker and smoker workers [39,40]. We evaluated the hypothesis that respiratory system entropy may be altered in these patients and that this analysis may be useful in the diagnosis of the mild abnormalities in these patients. It was performed under real medical conditions, investigating the general group of patients and specific groups of non-smoker and smoker patients. Three major findings were obtained: (1) initially, it was observed that exposition to asbestos introduced a complexity loss; (2) exposition associated with smoking also resulted in a complexity loss; and (3) entropy analysis accurately detected the early respiratory alterations in these patients. The present study extends our knowledge of entropy in respiratory diseases and adds to previous studies by providing evidence that a novel entropy-based index may be a sensitive indicator of future interstitial asbestosis.

### 4.1. Biometric and Pulmonary Function

The anthropometric characteristics of the subjects in the respiratory impedance (Table 1) and airflow (Table 3) entropy studies presented only small non-significant differences. Thus, the control groups and exposed groups can be considered homogeneous. 

Considering the spirometric changes, Table 1 and Table 3 show that the exposed individuals in both studied groups had lower mean values of FVC (absolute values) and FEV_1_ (absolute values and percentage of predicted values). When the FEV_1_ values were corrected for the expired volume (FEV_1_/FVC), there were no significant differences between groups. This result shows that exposure to asbestos caused a volume reduction (restrictive abnormality) but not airflow obstruction. These changes are in agreement with expectations in workers exposed to asbestos [13] and may be due to parenchymal involvement associated with asbestos fibres. It is important to point out that these changes are still within the normal range (31).

When exposed subjects were divided into smokers and non-smokers (Table 2 and Table 4), the appearance of obstructive phenomena in the smoker group was noted, as highlighted by the reduction of FEV_1_/FVC and FEF/FVC ratios. When the plethysmographic parameters were analysed, there was a clear relationship with the spirometric parameters, that is, an increase in RV and TLC (absolute values), and especially RV/TLC ratio. The increase in this last variable denoted air trapping, which is a characteristic phenomenon of bronchial obstructive syndrome. These changes are typical of smokers and likely reinforce the changes due to the exposition to asbestos.

### 4.2. Effects of Exposition to Asbestos in the Complexity Analysis

In agreement with previous studies in asthmatics with normal espirometric exams using approximate entropy in the analysis of the airflow [7] and respiratory impedance [18] patterns, the current study did not show significant differences in the comparative analysis of SampEnZrs, RPDEnZrs and SampEnV’ in controls and the general group of patients and non-smoking asbestos-exposed patients with normal espirometry (Figure 1a,b and Figure 2a). These results were likely related to the small physiological changes present in the studied sample. This sample may be characterized as early disease, and the mean changes observed in Table 1 and Table 3 are only suggestive of restriction as they were still within the normal range [41]. This outcome, however, is contradictory to the results of Dames et al. [17], who found a significant reduction in sample entropy in COPD patients with normal spirometric exams. A possible explanation for this discrepancy might be associated with the different biomechanical characteristics of the studied diseases; while asbestosis is characterized by restrictive changes, COPD is related to obstructive abnormalities. 

The most interesting finding in these comparisons was the significant reduction observed in RPDEnV’ (Figure 2b) in all of the studied asbestos exposed groups. Unfortunately, there are no data in the literature describing the early effects of restrictive diseases on respiratory entropy. In comparison to obstructive diseases, this finding is contradictory to previous studies, which have suggested that entropy analysis is unable to discriminate controls and patients with spirometric exams in the normal range [7,18]. However, it is in close agreement with the loss of complexity hypothesis [42] and provides evidence that the airflow pattern complexity is reduced in workers exposed to asbestos. Several factors could explain this observation. First, diffuse interstitial pulmonary fibrosis resulting from asbestos exposure is associated with a restrictive pattern in addition to a decreased flow and volume [43]. Another possible explanation for this phenomenon is an impairment of small airways [43] and gas exchange [44].

Comparing this finding with those using other techniques, this result confirms previous studies using plethysmographic analysis [45,46] and pulmonary diffusion [44], thus providing additional evidence that workers exposed to asbestos, but without a clinical diagnosis of asbestosis, may have substantially abnormal lung function. This result is also in line with previous authors who noted that respiratory abnormalities are detected in 80% of cases of significant exposition to asbestos, even before the observation of radiographic abnormalities [43].

### 4.3. Effect of Exposition to Asbestos Associated with Smoking in Complexity Analysis

In the biomechanical point of view, the smoking habit delays pulmonary clearance of asbestos fibers and may contribute to the severity and progression of asbestosis [39,40]. The comparison of non-smoking and smoking asbestos-exposed patients did not showed significant changes in SampEnZrs, RPDEnZrs and RPDEnV’ (Figure 1a,b and Figure 2b, respectively). In contrast, SampEnV’ was significantly reduced in smoker patients (Figure 2a). This is consistent with the cited physiological changes, and in accordance with recent studies of COPD patients that also found significant reductions in SampEnV’ in smokers with normal spirometric evaluations [17]. These results are also in agreement with those recently obtained by Raoufy et al. [19], who observed a significant reduction in the SampEn of the inter-breath interval in controlled atopic asthma, an obstructive disease. Also in line with Figure 2a, Arcentales et al. [47] showed decreased RPDEn in COPD patients, suggesting a lower respiratory system complexity, especially with regard to mechanical loading and maintenance of ventilation under high metabolic rates. These results provide further support for the loss of complexity hypothesis [42] and additional evidence that SampEnV’ may be useful as a novel index able to discern the adverse impacts of smoking. 

### 4.4. General Discussion

Breathing is a complex rhythmic motor act created by integrating the different inputs to the respiratory centres in the brainstem. These inputs are from peripheral and central chemoreceptors, the chest wall and pulmonary receptors, vagal afferents and non-respiratory central mechanisms [48]. As mentioned previously in the literature [49], changes in respiratory mechanics are additionally important factors for the genesis of the respiratory rhythm. Taking into consideration its complex nature, which includes various feedback/coupling interactions from both internal and external sources, the analysis of nonlinear dynamics seems to be an interesting way to characterize respiratory patterns [1,2,3,4,50,51,52,53]. 

In this context, problems with structural components and/or the connections among subsystems may lead to a loss of system complexity [27,54], which may be related to modifications in feedback mechanisms [54]. Goldberger [42] conceptualized the loss of system complexity as a general mechanism in ageing and disease. A pivotal role of this hypothesis in respiratory medicine is the concept that temporal fluctuations originate from the non-equilibrium of the respiratory system and often carry information about the process underlying such imbalances [55]. As a result, the analysis of fluctuations in the temporal behaviour of the respiratory variables may help to identify physiological changes, contributing to obtain new insights into the mechanism underlying the disease [56] and helping classify patients [5].

Previous studies have suggested that in the presence of more regular patterns of physiological biorhythms, the human body is less adaptive to stress and disease [57]. Another important hypothesis previously raised was that a greater signal regularity (smaller entropy) may also indicate an increased physiological system isolation, which is less adaptive to daily life demands [58]. This concept has been used in the area of cardiology, in which the study of heart rate variability is used to evaluate heart health. Heart rate variability reflects the ability to adapt to unpredictable stimuli [59], detecting and responding quickly to different circumstances.

Taking these two hypothesis into consideration, the reduction in SampEnV’ observed in Figure 2a and in RPDEnV’ observed in Figure 2b may be interpreted as a reduction of the ability of the patient’s respiratory system to meet increasing metabolic demands, associated with the presence of usual external daily activities. The presence of dyspnoea when performing physical efforts is a well-known symptom in workers exposed to asbestos. Therefore, the findings of this study are in close agreement with the loss of complexity [42] and reduction of adaptability to stress [57] in the presence of disease, providing further support to these hypothesis.

### 4.5. Diagnostic Use

The value of entropy analysis as a diagnostic tool in asbestos-exposed workers was evaluated using ROC curves (Figure 3a, Figure 4a, and Figure 5a). Consistent with the literature [18], this research revealed that SampEnZrs and RPDEnZrs did not present adequate diagnostic accuracy to identify early respiratory changes. This occurred in both the general group of patients and in specific groups of nonsmokers and smokers (Table 6, Table 7 and Table 8, respectively). Similarly, SampEnV’ did not achieve adequate diagnostic accuracy value, in contrast to earlier findings in COPD [17].

The results described in Table 6 and Table 7 and Figure 3 and Figure 4 suggested that RPDEnV’ could differentiate the general group of patients and the specific group of non-smoking asbestos-exposed patients from controls, indicating that this parameter is a potential marker for the initial changes in asbestosis. In smoker patients, SampEnV’ and RPDEnV’ presented adequate accuracy (Table 8). After the use of the more restrictive criteria associated with LOOCV analyses, RPDEnV’ and SampEnV’ continued to present an adequate value for the diagnostic accuracy. This was another important finding of this study, providing further support to the idea that these parameters may be useful in clinical applications [8]. In the general group of patients, the comparative analysis of the AUCs (Figure 3b), revealed that RPDEnV’ was significantly more accurate than SampEnV’ and RPDEnZrs, and it presented a non-significant increase in accuracy in comparison to SampEnZrs. The elucidation of this difference in diagnostic performance is a work in progress. In specific groups of non-smoking and smoking patients these differences were, in general, non-significant.

It is important to bear in mind that our sample consisted of individuals in the early stages of asbestosis. Thus, the changes observed in these patients were very small and difficult to detect. It is possible that several small changes in different respiratory subsystems are present in this initial phase. Hence, it could conceivably be hypothesized that RPDEnV’ could be more adequate to reflect the integrated effect of these changes than the other studied parameters.

SampEn and RPDEn are regularity indexes, and changes in these indexes do not necessarily represent changes in complexity. Being more irregular could relate to more random behavior, which is highly unpredictable but not complex in the structural sense, admitting a very simple description [60]. It is currently thought that the loss of complexity is related to a reduction in the capacity of the studied system to generate adaptive responses to stressors, in the number of functional structural components and in the coupling among these components [60,61,62,63]. In the particular case of the present study, the association of the observed reduction in entropy with the loss of complexity is justified by the fact that exposition to asbestos is characterized by structural changes in the respiratory system and its interconnections. In addition, these patients present a significant reduction in respiratory system adaptive responses to the stressors observed in daily life activities. From the biomechanical point of view, these stresses are associated with the increase in metabolism related with small exercises like climbing stairs, carrying bags etc. This completes the necessary requirements so that the observed reduction in entropy can be interpreted as a reduction in the complexity of the respiratory system. It is interesting to note that the observed reduction in entropy is consistent with previous results in asthma [18,19] and chronic obstructive pulmonary disease [17,47], which are also associated with a reduction in the adaptive responses, functional components and coupling. This provides additional evidence that respiratory diseases are consistent with the loss of complexity hypothesis.

### 4.6. Study Limitations

Finally, some potential limitations should be acknowledged. First, the present study included a relatively small sample size. This limitation was minimized by the LOOCV procedure, but it is still a limitation of this study, and future studies should include a larger number of subjects. The obtained results are valid only within the studied groups. In order for these results to be used in other groups of patients, the new patient population must be comparable in terms of biometric characteristics. Interested readers may examine the biometric characteristics and inclusion and exclusion criteria adopted to evaluate if they are likely to obtain similar outcomes in their own patient population. Only a preliminary evaluation of the entropy analysis in the identification of the adverse effects of smoking was performed. The promising results obtained in Figure 2a prompts future research to confirm these findings. It is known that sample entropy is susceptible to the choice of m and r, as well as the sampling rate used for data acquisition and epoch duration [25,30]. In the present study, SampEn was calculated using m = 2% and r = 20% of the SD of the studied time series, following the recommendations of Pincus and Goldberger [30]. As previously described [25], standardizing this choice of parameters was also important because it allowed us to compare different studies [7,17,18]. However, it could be argued that it is also a limitation since the parameters for SampEn estimation can be optimized [64,65], and this procedure was not used in the present study. The search for optimized parameters for entropy analysis of respiratory impedance and airflow pattern is a clear direction for future research. The comparative analysis of the ability of different entropy metrics to describe important physiological phenomena is a major area of interest [66]. The present investigation only examined SampEn and RPDEn, and other promising measures of entropy may also provide adequate diagnostic tool and were not explored. For example, dispersion entropy [67] and permutation entropy [68,69] are based on Shannon entropy. These promising entropy metrics presents a different operation principle from that of the SampEn used in this study, which was based on conditional entropy. Fuzzy approximate entropy [65], conditional entropy [70] and distribution entropy [71] are other potentially useful entropy measures. This hypothesis warrants further study. 

## 5. Conclusions

The aim of the present research was to examine the changes in respiratory impedance and airflow pattern complexity in patients exposed to asbestos. This study showed that exposition to asbestos introduced a complexity loss, as shown by a decrease in RPDEnV’, in non-smoker and smoker patients. Another interesting finding was that the SampEnV’ was reduced in smoker patients in comparison with non-smokers, suggesting a further complexity loss. One of the more significant findings of this study was that RPDEnV’ accurately detected the initial alterations in the general group of patients and in the specific groups of non-smokers and smokers. SampEnV’ also presented adequate diagnostic accuracy in patients exposed to asbestos and smokers. Taken together, these results suggest that entropy analysis might be an early and sensitive functional indicator of future interstitial asbestosis. A natural progression of this work is to confirm these findings in groups with larger sample sizes. 

## Figures and Tables

**Table 1:** Biometric and spirometric parameters of the studied groups in the impedance analysis. Significant changes are described in bold.

	Control (n = 34)	Exposed (n = 34)	p
Biometric data			
Age (years)	59.8 ± 13.1	64.0 ± 8.17	0.06
Weight (kg)	75.6 ± 13.1	76.8 ± 14.7	0.76
Height (cm)	164.3 ± 7.85	163.6 ± 10.08	0.75
BMI (kg/m^2^)	28.4 ± 4.65	28.6 ± 4.47	0.86
Spirometry			
FVC (L)	3.24 ± 0,83	2.80 ± 0.66	**<0.02**
FVC (%)	93.6 ± 13.7	88.1 ± 16.2	0.07
FEV_1_ (L)	2.59 ± 0.76	2.06 ± 0.48	**<0.003**
FEV_1_ (%)	92.4 ± 13.6	82.4 ± 14.7	**<0.006**
FEV_1_/FVC	78.4 ± 6.17	74.4 ± 10.6	0.17
FEF_25–75%_ (L)	2.39 ± 0.94	1.87 ± 0.85	**<0.02**
FEF_25–75%_ (%)	86.6 ± 25.1	76.4 ± 36.0	0.18
FEF/FVC	82.7 ± 22.3	89.2 ± 43.6	0.44
Forced oscillation			
Zm (cmH_2_O/L/s)	3.93 ± 1.17	5.00 ± 1.93	**<0.02**

Values are presented as the mean ± standard deviation; Significant correlations are presented in bold; FVC (L): forced vital capacity (litres); FVC (%): forced vital capacity (percentile values); FEV_1_ (L): forced expiratory volume in the first second (litres); FEV_1_ (%pred): forced expiratory volume in the first second (percentile of the predicted values); FEF_25–75%_: forced expiratory flow between 25% and 75% of the FVC; Zm: respiratory impedance modulus. Table 2 shows the effects of smoking on the exposed subjects. Significant changes were seen in spirometric indexes of small airway obstruction (FEF_25–75%_) and the plethysmographic indexes of residual volumes (RV).

**Table 3:** Biometric and spirometric parameters of the groups evaluated in the airflow pattern entropy study. Significant changes are described in bold.

	Control (n = 34)	Exposed (n = 30)	p
Biometric data			
Age (years)	60.9 ± 11.7	63.8 ± 8.25	0.36
Weight (kg)	73.4 ± 12.1	76.7 ± 14.5	0.38
Height (cm)	163.3 ± 8.43	164.6 ± 9.90	0.72
BMI (kg/m^2^)	27.8 ± 4.81	28.3 ± 4.55	0.65
Spirometry			
FVC (L)	3.15 ± 0.86	2.84 ± 0.66	**<0.03**
FVC (%)	93.8 ±15.4	87.4 ± 17.1	0.10
FEV_1_ (L)	2.51 ± 0.77	2.08 ± 0.48	**<0.004**
FEV_1_ (%)	93.1 ± 15.2	81.4 ± 15.0	**<0.002**
FEV_1_/FVC	78.2 ± 5.78	74.0 ±10.1	0.09
FEF_25–75%_ (L)	2.32 ± 0.95	1.98 ± 1.27	0.30
FEF_25–75%_ (%)	90.5 ± 28.3	73.2 ± 33.8	0.83
FEF/FVC	83.9 ± 25.1	84.9 ± 42.4	0.94
Forced oscillation			
Zm (cmH_2_O/L/s)	3.92 1.46	4.83 2.16	0.08

Values are presented as the mean ± standard deviation; significant correlations are presented in bold; FVC (L): forced vital capacity (litres); FVC (%): forced vital capacity (percentile values); FEV_1_ (L): forced expiratory volume in the first second (litres); FEV_1_ (%pred): forced expiratory volume in the first second (percentile of the predicted values); FEF_25–75%_: forced expiratory flow between 25% and 75% of the FVC. Zm: respiratory impedance modulus.

**Table 4:** Effects of smoking in exposed subjects in the airflow study. Significant changes are described in bold.

	Non-Smokers (n = 18)	Smokers (n = 12)	p
Spirometry			
FVC (L)	2.68 ± 0.74	3.11 ± 0.48	0.16
FVC (%)	85.3 ± 13.7	92.3 ± 21.3	0.40
FEV_1_ (L)	2.08 ± 0.53	2.04 ± 0.45	0.83
FEV_1_ (%)	83.5 ± 13.9	77.2 ± 15.7	0.26
FEV_1_/FVC	78.2 ± 7.38	66.0 ± 11.6	**<0.002**
FEF_25-75%_ (L)	2.15 ± 0.80	1.82 ± 2.01	**<0.05**
FEF_25-75%_ (%)	88.3 ± 33.8	50.9 ± 28.8	**<0.004**
FEF/ CVF	105.1 ± 41.6	53.6 ± 32.3	**<0.002**
Plethysmography			
RV (L)	1.97± 1.01	2.83 ± 1.04	**<0.02**
RV (%)	122.4 ± 51.4	154.2 ± 82.6	0.66
TLC (L)	4.73 ± 1.31	5.90 ± 0.91	**<0.02**
TLC (%)	100.5 ± 20.0	112.7 ± 31.3	0.43
RV/TLC (L)	38.3 ± 11.1	47.1 ± 10.3	**<0.04**
Raw (cmH_2_O/L/s)	5.98 ± 11.4	5.11 ± 5.19	0.80
Raw (%)	445.1 ± 855.0	368.8 ± 426.0	0.77
sGaw (L/s/cmH_2_O/L)	0.25 ± 0.41	0.09 ± 0.07	0.20
sGaw (%)	98.6 ± 156.2	50.2 ± 38.5	0.30
Forced oscillation			
ZmV’ (cmH_2_O/L/s)	4.74 ± 2.01	4.97 ± 2.43	0.77

Values are presented as the mean ± standard deviation; FVC (L): significant correlations are presented in bold; forced vital capacity (litres); FVC (%): forced vital capacity (percentile values); FEV_1_ (L): forced expiratory volume in the first second (litres); FEV_1_ (%pred): forced expiratory volume in the first second (percentile of the predicted values); FEF_25–75%_: forced expiratory flow between 25% and 75% of the FVC; RV: residual volume (litres); TLC: total lung capacity; raw: airway resistance; sGaw: specific airway resistance; Zm: respiratory impedance modulus.

**Table 5:** Spearman’s correlation coefficients between entropy analysis and pulmonary function exams. Significant associations are presented in bold.

		SampEnV’	RPDEnV’	SampEnZrs	RPDEnZrs
FEV_1_ (%)	r	0.10	0.15	-0.12	0.21
	p	0.42	0.24	0.32	0.08
FVC (%)	r	0.04	0.15	-0.20	0.02
	p	0.74	0.42	0.09	0.89
FEF_25-75%_ (%)	r	0.23	0.01	0.03	**0.26**
	p	0.07	0.93	0.78	**<0.03**
Zrs (cmH_2_O/L/s)	r	−0.10	0.01	**−0.33**	**−0.26**
	p	0.40	0.93	**<0.006**	**<0.04**

SampEnV’: sample entropy of the airflow signal; RPDEnV’: recurrence period density entropy of the airflow signal; SampEnZrs: sample entropy of the respiratory impedance signal; RPDEnZrs: recurrence period density entropy of the respiratory impedance signal.

**Table 6:** Evaluation of the diagnostic use of respiratory impedance and airflow pattern complexity in the detection of the initial respiratory changes in asbestos-exposed workers, including non-smokers and smokers patients.

	SampEnZrs	RPDEnZrs	SampEnV’	RPDEnV’
**AUC**	0.633	0.522	0.507	0.766
**Se (%)**	56.7	46.7	50.0	70.0
**Sp (%)**	64.7	47.1	50.0	64.7
**Cut-off**	>0.505	≤0.621	≤0.213	≤0.446

Area under the receiver-operating characteristic curve (AUC), sensitivity (Se), specificity (Sp) and respective cut-off points.

**Table 7:** Diagnostic accuracy of the entropy of respiratory time series in patients exposed to asbestos and non-smokers.

	SampEnZrs	RPDEnZrs	SampEnV’	RPDEnV’
**AUC**	0.656	0.540	0.625	0.796
**Se (%)**	77.3	63.64	77.8	88.9
**Sp (%)**	55.9	58.8	50.0	58.8
**Cut-off**	>0.451	>0.626	>0.212	≤0.470

Area under the receiver-operating characteristic curve (AUC). sensitivity (Se). specificity (Sp) and respective cut-off points.

**Table 8:** Diagnostic accuracy of the entropy of respiratory time series in patients exposed to asbestos and smokers.

	SampEnZrs	RPDEnZrs	SampEnV’	RPDEnV’
**AUC**	0.596	0.645	0.706	0.721
**Se (%)**	66.7	58.3	75.0	66.7
**Sp (%)**	58.8	58.8	64.7	64.7
**Cut-off**	>0.468	≤0.600	≤0.195	≤0.443

Area under the receiver-operating characteristic curve (AUC), sensitivity (Se), specificity (Sp) and respective cut-off points.
